# Research Progress on Polymer Solar Cells Based on PEDOT:PSS Electrodes

**DOI:** 10.3390/polym12010145

**Published:** 2020-01-07

**Authors:** Lin Hu, Jiaxing Song, Xinxing Yin, Zhen Su, Zaifang Li

**Affiliations:** China-Australia Institute for Advanced Materials and Manufacturing (IAMM), Jiaxing University, Jiaxing 314001, China; hulin@zjxu.edu.cn (L.H.); songjx@zjxu.edu.cn (J.S.); xxyin@zjxu.edu.cn (X.Y.); suzhen@zjxu.edu.cn (Z.S.)

**Keywords:** polymer solar cells, flexible electrodes, PEDOT:PSS, electrical conductivity

## Abstract

Solution-processed polymer solar cells (PSCs) have attracted dramatically increasing attention over the past few decades owing to their advantages of low cost, solution processability, light weight, and excellent flexibility. Recent progress in materials synthesis and devices engineering has boosted the power conversion efficiency (PCE) of single-junction PSCs over 17%. As an emerging technology, it is still a challenge to prepare solution-processed flexible electrodes for attractive flexible PSCs. Poly(3,4-ethylenedioxythiophene): poly(styrenesulfonate) (PEDOT:PSS) is one of the most promising candidates for electrodes due to its high conductivity (>4000 S/cm), excellent transmittance (>90%), intrinsically high work function (*W*_F_ > 5.0 eV), and aqueous solution processability. To date, a great number of single-junction PSCs based on PEDOT:PSS electrodes have realized a PCE over 12%. In this review, we introduce the current research on the conductive complex PEDOT:PSS as well as trace the development of PEDOT:PSS used in electrodes for high performance PSCs and perovskite solar cells. We also discuss and comment on the aspects of conductivity, transmittance, work-function adjustment, film preparing methods, and device fabrications. A perspective on the challenges and future directions in this field is be offered finally.

## 1. Introduction

Solution-processed polymer solar cells (PSCs) have attracted dramatically increasing attention over the past few decades owing to their advantages of low cost, easy fabrication, light weight, and good flexibility. Recent achievements in materials development and devices engineering have produced a record power conversion efficiency (PCE) over 17% [1], demonstrating an anticipated application prospect. However, a high-quality electrode is another challenge for high performance PSCs, especially the flexible electrode for the corresponding flexible devices.

It is well known that a typical PSC configuration consists of a light absorption layer sandwiched between two electrodes (the top one and the bottom one). At least one of them has to be transparent for light illumination through and then to the light absorption layer. Device performance of PSCs is highly dependent on the quality of the electrodes, and a stricter requirement is necessary in the case of flexible electrodes. A high-quality flexible electrode should possess characteristics such as superior mechanical flexibility, low sheet resistance, high transmittance in the visible-light range, and excellent thermal stability. Among current candidates, indium-tin-oxide (ITO) and vacuum-deposited metals-based flexible electrodes have been commercially used in PSCs. However, high production costs and especially poor mechanical stability strongly hinder their practical applications [2]. Therefore, many scientific communities are searching other candidates including silver nanowires (AgNWs), silver-grids, conductive polymers (Poly(3,4-ethylenedioxythiophene): poly(styrenesulfonate); PEDOT:PSS), and carbon-based materials, etc.

**Figure 1:**
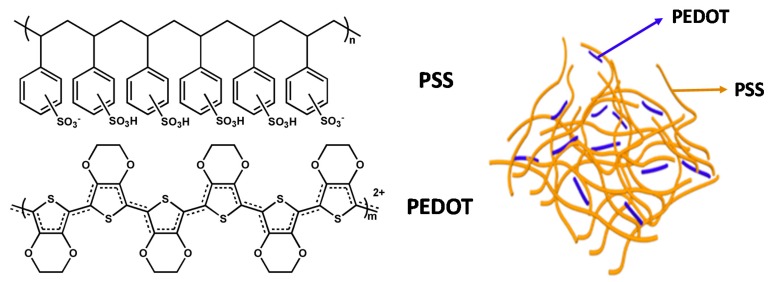
The chemical structure and schematic core-shell structure of Poly(3,4-ethylenedioxythiophene): poly(styrenesulfonate) (PEDOT:PSS).

The commercially available PEDOT:PSS is one of the best choices for flexible electrodes owing to its high transmittance in the visible range, high and adjustable conductivity, intrinsically high work function, excellent thermal stability, and good film-forming capability as well as superior mechanical flexibility. PEDOT:PSS (Figure 1) is composed of positively charged conjugated PEDOT and negatively charged saturated PSS. Its aqueous dispersion can be processed to form a thin film on either rigid or flexible substrates by various solution-processing techniques. The achieved PEDOT:PSS film is smooth and ideal for the application of electrodes in electronics. PEDOT:PSS exhibits a wide range of conductivities from 10^−4^ to 10^3^ S/cm^−1^, determined by synthetic conditions, doping additives, or post-treatment methods. PEDOT:PSS film also possesses a high work function of 5.0–5.2 eV, matching well with the highest occupied molecular orbital (HOMO) level of most of the polymer donors in the absorption layer. The matched energy level ensures that PEDOT:PSS is a suitable electrode for hole collection. Dedoping of PEDOT:PSS can result in reduction of the *W*_F_, endowing potential fabrication of low-work function PEDOT:PSS electrodes for electron collection. In 2002, Zhang et al. [3] first demonstrated that the sorbitol-doped PEDOT:PSS with an appropriated conductivity and work function could be employed as transparent electrodes in PSCs. Since then, more and more scientists have introduced and optimized PEDOT:PSS as a flexible electrode material. A high PCE over 12% for single-junction PSCs based on PEDOT:PSS electrodes has been achieved by several groups [4,5].

In this review, we describe the basic synthesis and properties of PEDOT:PSS complex. Comments and discussions are offered on the conductivity, transmittance, work-function adjustment, as well as processing technologies of the PEDOT:PSS films. Then, we focus on the applications of PEDOT:PSS films as electrodes for PSCs. Various fabrication technologies and the application of PEDOT:PSS used in bottom, top, and both electrodes are all surveyed. A perspective on the challenges and future directions in this field is offered finally.

## 2. PEDOT:PSS Electrodes

### 2.1. Synthesis and Commercialization of PEDOT:PSS Complex

A very feasible way to obtain PEDOT:PSS complex is the oxidative polymerization of EDOT in aqueous dispersion using sodium peroxodisulfate as the oxidant combined with a PSS template polymer. The PSS in the complex has two important roles. One is to act as counterions for charge balancing. A monomolecular oxidation product can be formed without counterions. Another role of PSS is to keep the PEDOT segments dispersed in the aqueous solution as the PEDOT polycation is hardly soluble in any solvent. The obtained PEDOT:PSS aqueous dispersion is a deep-blue opaque solution. We also need to note that the molecular weight of PEDOT is only among 1000 to 2500 Da (about 6 to 18 repeating units) resembling an oligomeric nature [6]. Research from Inganas, et al. demonstrated this polymer complex is stable due to the ionic PEDOT^+^ and PSS^−^ not being separated by capillary electrophoresis [7].

PEDOT:PSS aqueous dispersion was first commercialized under the trade name of Baytron^®^ by Bayer AG, followed by H.C. Starck and currently by Heraeus under the trade name of Clevios^™^. The company of Agfa Gevaert N.V. (German) also introduced PEDOT:PSS for large-scale printing applications under the trade name of Orgacon^™^. The PEDOT:PSS dispersions are classified into different grades based on the solids content and the ratio of PEDOT to PSS. The component strongly influences the particle size and distribution, viscosity, conductivity, and transmittance after film formation of the dispersion [6]. Table 1 summarizes important properties of the commercial PEDOT:PSS Clevios dispersions.

### 2.2. Properties of PEDOT:PSS

PEDOT:PSS possesses several characteristics, such as high transmittance in the visible range, high and adjustable electrical conductivity, excellent thermal stability, high work function, as well as good film-forming ability by versatile fabrication techniques. These unique features ensure its widespread applications in various photo-electronic devices.

The transmittance of the PEDOT:PSS electrode is highly dependent on the film thickness. Generally, PEDOT:PSS thin films with a thickness around 100 nm possess high transmittance over 90% at 550 nm. The absorption spectrum of PEDOT:PSS is almost identical to that of in situ chemically polymerized PEDOT without PSS. Moreover, the addition of PSS or other additives like high boiling solvents does not influence the optical properties significantly. However, PEDOT:PSS film treated by concentrated sulfuric acid demonstrates a slight reduction in the transmittance [10]. The reason is mainly attributed to a stacking conformational change of the conjugated ionic PEDOT^+^ segments caused by the significant loss of PSS in the processing.

**Figure 2:**
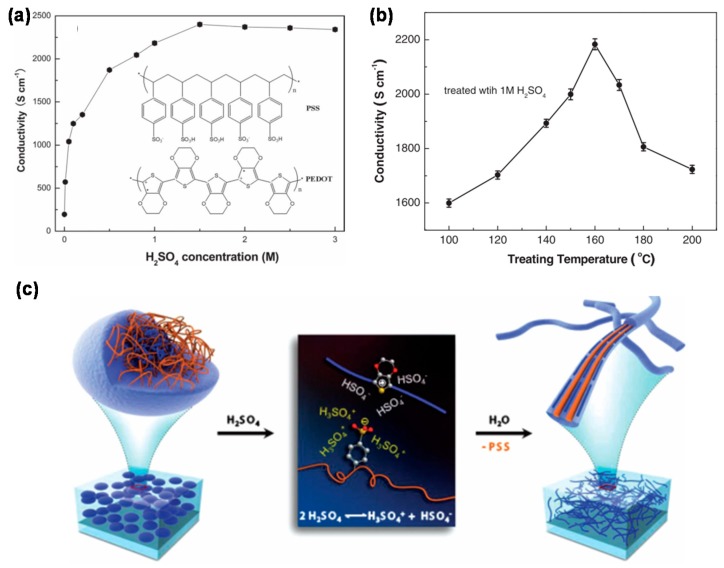
Conductivities of PEDOT:PSS films after treatment with H_2_SO_4_ solutions [20]. (**a**) PEDOT:PSS films treated with H_2_SO_4_ solutions of various concentrations at 160 °C. (**b**) PEDOT:PSS films treated with 1 mol/L H_2_SO_4_ at various temperatures. The PEDOT:PSS films in (**a**,**b**) were treated with 1 mol/L H_2_SO_4_ only once. (**c**) Diagram of the structural rearrangement of PEDOT:PSS via a concentrated H_2_SO_4_ treatment [16].

Electrical conductivity is one of the most important parameters considering its applications in various devices such as electrodes. The electrical conductivity is strongly dependent on the film morphology, chemical and physical structure, as well as the composite ratio, which in turn can be strongly modified via a variety of post treatments. In the past few decades, many efforts have been made in improving the electrical conductivity of PEDOT:PSS, and several approaches such as second doping as well as post-treatment have been reported to significantly improve the conductivity of PEDOT:PSS. In 2002, Kim et al. reported the electrical conductivity enhancement of PEDOT:PSS by doping polar organic solvents [11]. They demonstrated that the electrical conductivity of PEDOT:PSS can be enhanced by one order when dimethylformamide (DMF) is added into the aqueous solution, while enhancement over two orders can be achieved by doping dimethyl sulfoxide (DMSO). Conductivity enhancement was also reported by employing other polar organic solvents like ethylene glycol (EG), glycerol, and sorbitol [3,12]. Moreover, anionic surfactant, ionic liquid, as well as nonionic surfactant were found to be very effective in improving the electrical conductivity of PEDOT:PSS [13,14,15]. In addition to doping, film post-treatment is another common and effective method for enhancing the conductivity of PEDOT:PSS. As for post-treatment, a solvent or solution is dropped (or vapored) onto a PEDOT:PSS film, or the film is dipped into a solvent or a solution. The conductivity can be obviously enhanced through a post treatment with an organic solvent like DMSO, EG, methanol, 2-methoxyethanol as well as co-solvent, aqueous or organics solutions of salts and acids [16,17,18,19]. The strong acids such as HCl and H_2_SO_4_ are the most often used method for acid treatment. Ouyang and colleagues for the first time observed that conductivity can be obviously improved through a post treatment of the PEDOT:PSS film with concentrated H_2_SO_4_ [20]. As shown in Figure 2, the electrical conductivity of PEDOT:PSS is highly related to the concentration of H_2_SO_4_ and treated temperature. The electrical conductivity of PEDOT:PSS film can be enhanced to 2400 S/cm treated by diluted 1.5 mol/L H_2_SO_4_ and up to 3100 S/cm by repeating the process. This electrical conductivity is comparable to that of commercialized indium tin oxide (ITO) electrodes. Later, Lee’s group improved the electrical conductivity by optimizing the post-treatment conditions with concentrated H_2_SO_4_ [16]. They found the conductivity increased as the improvement of PEDOT:PSS crystallinity and a high conductivity up to 4380 S/cm was obtained. Up to now, the highest conductivity has reached 4840 S/cm through this H_2_SO_4_ post treatment method [21]. The H_2_SO_4_-treated PEDOT:PSS demonstrates unique metallic or semimetallic behavior, indicating strong interactions among the PEDOT chains. Although great progress has been made in the improvement of electrical conductivity, its value is still much lower than that of metal electrodes. In addition, the conductive mechanism is still controversial and further efforts are necessary for exploring the origins of this conductivity enhancement.

### 2.3. Fabrication Technologies of PEDOT:PSS Electrodes

**Figure 3:**
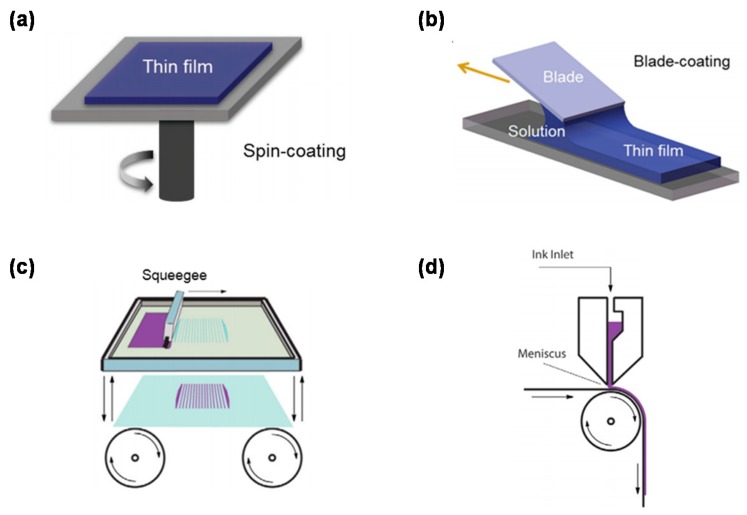
Schematic of (**a**) spin coating, (**b**) doctor blade coating, (**c**) screen printing, and (**d**) inkjet printing [22].

PEDOT:PSS can be deposited and can readily form continuous thin film on either rigid or flexible substrates by all common techniques employed for the deposition of waterborne coatings (such as spin coating, doctor blading, screen printing, inkjet printing; see Figure 3) [22]. However, pristine PEDOT:PSS solution possesses hydrophobic nature and cannot be processed directly. One of the effective methods is to employ plasma or ozone cleaning, but this makes the process complex. Another preferable method is to add surfactants (such as Zonyls, Dynols, and Triton X-100) into PEDOT:PSS solution to improve its wetting property [23,24,25]. Previous reports demonstrate that the contact angle of commercially available PEDOT:PSS solution (PH1000) on top of active layer can be reduced from 99.6° to 29.1° by doping 0.4% surfactant PEG-TmDD [14]. The PEDOT:PSS films achieved by the above procedures are smooth, and the thickness can also be controlled from several to hundreds of nanometers, making these films attractive as cladding layer for thin film device applications.

Spin coating is the most commonly used method in the lab to prepare a highly conductive PEDOT:PSS film as electrodes in the PSCs. During the preparation, the PEDOT:PSS dispersion is dropped onto a substrate followed by a substrate spinning at a selected speed. The thickness mainly depends on the solution concentration and the spinning speed. Normally, the PEDOT:PSS electrodes achieved by spin coating methods possess the film thicknesses of 100–200 nm and transmittances of over 80% in the wavelength range of 350–600 nm, which is about 10% lower than that of commercialized ITO electrodes beyond 600 nm [8]. However, this technique is unsuited to a device with a large area and not compatible with the industrial roll-to-roll processing. Moreover, more than 80% of the solution/ink flies away during the processing. This motivates intensive research on printing techniques. The doctor blading technique is another coating technique that can be integrated with the roll-to-roll processing. The coating solution is placed in front of a sharp blade, and a thin wet film is formed after the blade moves across the substrate. The distance is typically 10–500 μm [26]. The coating solution is placed in front of a sharp blade, and a thin wet film is formed after the blade moves across the substrate. Film thickness depends on the concentration, viscosity of the solution, and the surface energy of the substrates. The doctor blading technique is not used as widely as the spin-coating method because it is time-consuming and requires a lot of materials to get the right conditions for film processing. Jang et al. propose a simple and fast patterning method based on the doctor blading by adjusting the wetting properties of PEDOT:PSS. They also reveal that the evaporation-induced flow exerts a great influence on the film morphology [27]. Screen printing is also a commonly used method. In this technique, a pre-patterned mesh is used to transfer ink onto a substrate. Then a blade or squeegee is moved across the screen to fill the open mesh apertures with ink followed by a reverse stroke to ensure the screen touches the substrate momentarily along a line of contact. Note that this technique requires a high viscosity of the solution, and the generated films always possess a large thickness. Therefore, this processing technique might not be used for fabricating the active layer but might be suitable for depositing PEDOT:PSS films.

Considering the difference in processing technology and concomitant film-forming mechanism, the requirements for characteristics including viscosity, density, and surface tension of the solution are different. Therefore, rational optimization of the solution composition, such as solvent and additives, is of great importance to obtain a high quality conductive film.

## 3. Polymer Solar Cells Based on PEDOT:PSS

Polymer solar cells (PSCs) have emerged as an alternative photovoltaic technology and attract tremendous attention owing to the advantages of a wide range of materials source, light weight, high mechanically flexibility, and solution processability. During a device preparation, ITO is always employed as the transparent electrode, and the vacuum-deposited metals (such as Al, Ag, Au) are generally used as top electrodes. Despite its good combination of high optical transmittance and high conductivity, ITO is too expensive and rigid while the vacuum-deposited metals consume high energy and require expensive deposition facilities with high-cost maintenance. Therefore, many scientific communities devote themselves to developing novel conductive materials as an alternative approach. The solution-processable PEDOT and its derivatives have become main candidates and have been functioned as bottom, top, and both electrodes in the PSCs (Table 2).

### 3.1. PEDOT:PSS as Bottom Electrode for Polymer Solar Cells

For bottom transparent electrode, to replace the traditional ITO, two material properties (transmittance in the visible spectral range and electrical conductivity) are the most relevant. The first attempt to use PEDOT:PSS as bottom transparent electrodes for PSCs was reported by Zhang et al. in 2002 [3]. They improved the conductivity of the PEDOT:PSS film by two orders of magnitude to 10 S/cm through mixing the original solution with glycerol or D-sorbitol. The transmittance of the optimized polymer electrode films was over 80% in the wavelength range of 350–600 nm with thicknesses of 150–200 nm. Ultimately, the device based on glass/PEDOT:PSS as the anode delivered a PCE of 0.36% as compared to 0.46% of the glass/ITO-based device. Though the PCEs of the cells were inferior, it demonstrated the feasibility of conducting polymer acting as the transparent electrodes for PSCs. Then Ouyang and co-workers further improved the conductivity of the PEDOT:PSS film to 155–160 S/cm by adding ethylene glycol (EG) or meso-erythritol into its dispersion [28]. The conductivity enhancement was attributed to the additive-induced conformational change in the PEDOT chains, which increased both intra-chain and inter-chain charge carrier mobility. A higher PCE of 1.5% under 100 mW cm^−2^ illumination for the PSCs was thus obtained. Since then, different strategies, such as adding high boiling polar solvents (DMSO etc.) and post treatment with acid (sulfuric acid, phosphoric acid, or organic acids) or surfactant, have been used for enhancing its conductivity to meet the requirement for practical electrode application [40,46,47]. Na et al. reported the average conductivity of pristine PEDOT:PSS (PH500) only possessed an average conductivity around 1 S/cm on both glass and PET substrates, while it can be improved to ~500 S/cm after adding 5% DMSO [29]. They optimized the PEDOT:PSS film with a thickness of 100 nm exhibiting a sheet resistance of 213 Ω/sq and transmittance above 90% in the visible wavelength range. By employing this highly conductive polymer film as bottom transparent electrode, they developed highly efficient ITO-free PSCs on both glass (PCE = 3.27%) and flexible plastic substrates (PCE = 2.8%) for the first time. Though the efficiencies were comparable to those of ITO-based devices on glass (PCE = 3.66%) and flexible substrates (PCE = 2.9%), the ITO-free PSCs on the flexible substrate manifested superior mechanical robustness. It is worth noting that a homogenous PEDOT:PSS layer was difficult to reproduce once DMSO was added. To overcome this problem, different surfactants were always added for a better adhesion and wettability. Ahlswede et al. reported a highly conductive PEDOT:PSS formulation consisting of four chemicals (including DMSO, diethylene glycol, sorbitol, and sulfonyl) to maintain adhesion, coverage, and conductivity at the same time [19]. Controlling the PEDOT:PSS film with a sheet resistance of 80 Ω/sq at a maximum transmittance of 74%, they obtained a PCE of 2.6% based on P3HT:PCBM active layer. For a higher conductivity, the PEDOT:PSS film can be post-treated with polar solvents or acids. Alemu et al. proposed a simple yet robust film treatment method with methanol to enhance the conductivity of PEDOT:PSS by four orders of magnitude to 1362 S/cm [30]. They prepared ITO-free PSCs with standalone PEDOT:PSS anodes showing almost equal performance to that of ITO electrodes. Sulfuric acid (H_2_SO_4_) treatments have been considered as very promising methods for PEDOT:PSS films with a high conductivity and transmittance. Ouyang and co-workers first reported that the conductivity of PEDOT:PSS can be enhanced to over 3000 S/cm via dropping H_2_SO_4_ solutions onto the pristine PEDOT:PSS films followed by thermal annealing [20]. Moreover, the dilute H_2_SO_4_ treatment slightly affects the transmittance of the PEDOT:PSS film. A 66 nm-thick film possessed a transmittance of 87% at 550 nm, and the transmittance was higher than 90% in the wavelength below 500 nm. The PSCs with the H_2_SO_4_-treated PEDOT:PSS film as anode exhibited comparable photovoltaic performance to that of the control devices using ITO anode. In addition to being used as an anode for holes collection in the device, PEDOT:PSS can also be modified as a cathode for electrons collection. Zhou and co-workers pioneered the work on utilization of PEI or PEIE to modify PEDOT:PSS and generate a low work function bottom electrode for ITO-free PSCs [41].

Incorporation of carbon materials and metal nanowires or grids into PEDOT:PSS is another effective approach to improve the conductivity of the PEDOT:PSS films for PSCs. Chen and co-workers demonstrated scalable and highly conductive PEDOT:PSS:CNTs (hc-PEDOT:PSS:CNTs; CNTs—carbon nanotubes) transparent electrode for high performance optoelectronics [31]. The composite electrode possessed a high conductivity of 3264.27 S/cm as well as a high transmittance over 85%. ITO-free PSCs based on glass/PEDOT:PSS:CNTs electrode achieved a PCE of 7.47% with high stability. Later, they also produced a composite electrode with low work function via in situ polymerization of the PEDOT:PSS-based sulfonated carbon nanotubes (SCNTs) as templates [34]. The resultant PEDOT:PSS:SCNT films (70 nm) possessed a work function of 4.4 eV and conductivity over 3500 S/cm as well as ~83% transmittance in the visible wavelength range. The electrodes were successfully integrated as a cathode in PSCs and perovskite solar cells with PCEs of 9.91% and 13.31%, respectively. Metal nanowires or grids have been shown to form high quality electrode coatings from solution processes. The electrodes have a sheet resistance and transmittance similar to those of common ITO. However, one of the major challenges in employing these metallic electrodes with nanostructures for thin-film electronic devices is their high surface roughness that create shorts or leakage across the semiconductor films. To address this issue, an inorganic–organic hybrid electrode consisting of metal nanowires or grids and PEDOT:PSS was developed. The polymer PEDOT:PSS could fill the gaps among nanowires or grids, and the film therefore possesses a smoothed surface as well as an increased conductivity. Zou et al. reported Ag grid/PEDOT:PSS hybrid transparent electrode could be used to replace ITO for the fabrication of inverted structure PSCs [32]. The performance of the devices could be tuned easily by varying the width and separation of the metal grids. PSCs fabricated using this hybrid electrode showed efficiencies as high as ~3.2%. Krebs and co-workers also demonstrated a multistack flexible electrode comprising a structure of polyethylene terephthalate (PET)/Ag grid/PEDOT:PSS can be employed as front electrodes for ITO-free all printed PSCs [48]. Subsequently, they further expanded this flexible electrode to fabricate large area, flexible organic tandem solar cell modules via roll-to-roll manufacture under ambient atmosphere [49]. Noh et al. reported the AgNWs/hcPEDOT hybrid electrodes could be prepared with a one-step spray-coating [50]. The film thickness, optical transmittance, and sheet resistance of the hybrid electrodes were easily controlled by varying the spray deposition time. With these one-step spray-coated hybrid electrodes on glass substrate, the device exhibited a PCE of 2.16% based on P3HT:PCBM active layer system.

**Figure 4:**
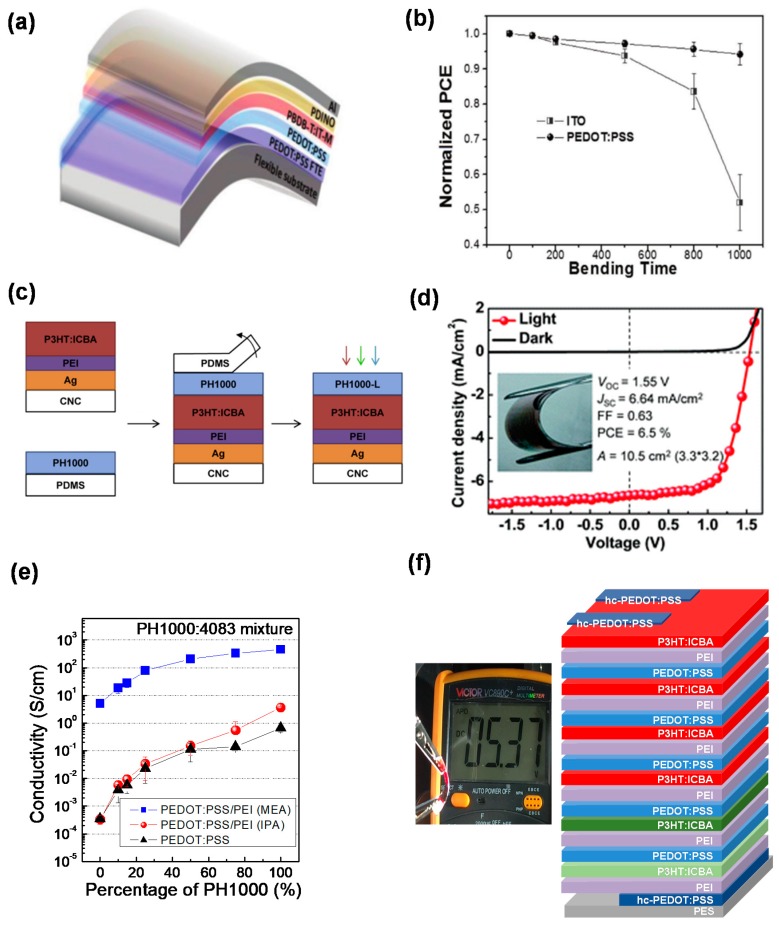
(**a**) Schematic architecture of highly efficient non-fullerene-based ITO-free PSCs and (**b**) decays of normalized PCEs of the corresponding devices in a continuous bending test [33]. (**c**) The fabrication procedure of recyclable solar cells on cellulose nanocrystal (CNC) substrates [59] and (**d**) flexible large-area organic tandem solar cells [60] using film-transfer laminated PEDOT:PSS as top electrode. (**e**) Conductivity tuning of the PEDOT:PSS/PEI interconnecting layer and (**f**) all-solution-processed all-plastic multijunction cells displayed *V*_OC_ of 5.37 V [63].

The above examples indicate the solution-processable PEDOT:PSS is a promising candidate material as bottom transparent electrode for ITO-free PSCs. Recently, benefiting from the progress of non-fullerene-based bulk heterojunction PSCs, more and more ITO-free PSCs with PEDOT:PSS as bottom transparent electrode have yielded a high PCE over 10% on both rigid glass and flexible substrates [33,51]. As shown in Figure 4a,b, the flexible PSCs maintain an excellent bending flexibility after 1000 cycles (a high retention ≈94% of the initial efficiency). Moreover, the PSCs are easy to realize in an all-solution-processed fabrication due to the avoidance of the sputtered ITO, which is compatible with various printing technologies for large-scale and flexible devices.

### 3.2. PEDOT:PSS as Top Electrode for Polymer Solar Cells

In common PSC devices, metals including MoO_3_/Ag or LiF/Al are used as the top electrodes. However, the thermal evaporation of metal electrodes requires high-vacuum system, which is complicated, expensive, and highly energy consuming. Moreover, thermal damage of interface may occur during metal evaporation. Therefore, solution-processed PEDOT:PSS top electrode is proposed due to its advantages of being fast, energy-efficient, cost-effective, and transparent [52]. There are two main challenges for fabricating high-quality PEDOT:PSS top electrodes. Firstly, commercially available high-conductivity PEDOT:PSS formulations (dispersed in aqueous solutions), such as PEDOT:PSS PH500 and PEDOT:PSS PH1000, exhibit poor wetting property on hydrophobic photoactive layers. Secondly, unlike inorganic solar cells, the photoactive layers of PSCs are thin (~100 nm) and soft. The photoactive layer is easily penetrated, leading to large leakage current or even shortage and poor device performance. Thus, a proper pattern is necessary.

To tune the photoactive layer surface from hydrophobic to hydrophilic for better deposition of aqueous PEDOT:PSS, many efforts have been made. One effective method is inserting an interlayer, by incorporating a thermally evaporated layer of lithium-doped bathophenanthroline (BPhen:Li) [53], an amphiphilic layer of PAH-D [54], or a low-conductivity PEDOT:PSS (Al4083 or CPP 105D) buffer layer with more surfactant [55] so that PEDOT:PSS top electrodes can be easily deposited. To simplify the device fabrication, Zhou et al. mixed CPP 105D with PH1000 to enable a single-step deposition of PEDOT:PSS top electrode [35]. Li et al. added a nonionic surfactant PEG-TmDD to PH1000, which can solve wetting problem and enhance the electrical conductivity to 526 S/cm [14]. Mao et al. reported a Maobi coating strategy in which PEDOT:PSS electrode can be fabricated from aqueous PH1000 mixed with EG and surfactant using Maobi as coating tool [36]. PSCs with a structure of glass/ITO/ZnO/PBDB-T:ITIC/PEDOT:PSS exhibit a high PCE of 7.38%. Mild plasma treatment is another efficient method to turn the surface to hydrophilic, but the photoactive layer could be damaged if this is not performed with proper processing techniques [56].

Considering the pattern issues of PEDOT:PSS top electrode, different methods have been developed with the popularity of the flexible electrode. Lim et al. fabricated PEDOT:PSS top electrode through spray coating and patterned through a shadow mask [57] Hau et al. and Zhou et al. used poly(dimethylsiloxane) (PDMS) to peel off part of the interlayer, leaving behind a patterned area. Then high-conductive PEDOT:PSS formulation was spin-coated to form top electrode [21,33]. However, this peeling-off method including solution process could damage layers underneath and bring difficulty in precisely controlling the pattern of the PEDOT:PSS film. Further, modified film-transfer lamination technique was reported by Wang et al. and Gupta et al. [37,58]. Generally, a PDMS stamp coated with PEDOT:PSS electrode was placed in close contact with the receiving surface. The PDMS stamp was then peeled off, leaving the transferred electrode in place. The application of film-transfer lamination method could solve the problem of damaging the layers underneath, since the PEDOT:PSS film is transferred dry. Zhou et al. fabricated PSCs with laminated PH1000 as top electrode on recyclable cellulose nanocrystal (CNC) substrates (Figure 4c) [59]. This dry film-transfer lamination process avoids swelling damage to the CNC substrate, since CNC can be easily dissolved in water. The PSCs exhibit a maximum PCE of 4.0% when illuminated through the semitransparent PEDOT:PSS top electrode. To break the Shockley–Queisser limit as well as achieve high efficiency, tandem solar cells have been investigated. Tong et al. first reported on organic tandem solar cells with PEDOT:PSS as the top electrode using film-transfer lamination method by PDMS medium. The fabricated devices exhibit an open-circuit voltage of 1.62 V and an average PCE of 3.6% [38]. Later, Mao et al. also employed this film-transfer lamination method to fabricate flexible large-area organic tandem solar cells with P3HT:ICBA as bottom cells and PTB7-Th:PCBM as top cells [60]. To reduce the series resistance, 80 nm silver grids were evaporated on top of the laminated PH1000 top electrode, yielding a PCE of 6.5% (Figure 4d). Yin et al. replaced PDMS with cheaper and more easily accessible plastic wrap as transfer medium [61]. PSCs based on this new medium exhibited an averaged PCE of 4.0%, which was comparable with PDMS method. In 2016, Li et al. reported micrometer-thick, highly conductive, free-standing PEDOT:PSS films with a high conductivity of 1400 Scm^−1^, which can be laminated as the top electrode directly without using PDMS medium [39]. By using this technique, the PEDOT:PSS films can be easily scaled up, which gives them great potential in large-area applications [42].

### 3.3. PEDOT:PSS as Both Bottom and Top Electrodes for Polymer Solar Cells

In light of the advantages of their low cost, low weight, and flexible manufacture, all-plastic organic solar cells with ITO-free, metal-free, and vacuum-free PEDOT:PSS electrodes are emerging. Hau et al. demonstrated the first all-plastic solar cell with PEDOT:PSS acting as both bottom cathode and top anode, showing an efficiency of 0.47% [40]. Zhou et al. developed a universal method to tune work function of conductors with PEI. Devices with a PEI-coated PH1000 bottom electrode and a PEDOT:PSS blend top electrode yielded an average PCE of 3.5% [41]. The same group further reported the first semitransparent all-plastic solar cell fabricated in ambient air through film-transfer lamination technique [43]. Li et al. treated PEDOT:PSS bottom electrode with H_2_SO_4_ and PEI solution through a two-step dipping process. A high conductivity of 1561 S/cm and a low work function of 4.0 eV can be obtained, yielding a champion efficiency of 4.0% [44]. To avoid the corrosion of H_2_SO_4_ on plastic substrates such as PET or PES, Meng et al. used a mild phosphoric acid (H3PO4) to treat the PEDOT:PSS electrode, and an enhanced conductivity of 1460 S/cm was acquired [45]. With the flexible electrode of PES/H_3_PO_4_-treated PEDOT:PSS, they fabricated flexible all-plastic solar cells (PES/H_3_PO_4_-treated PEDOT:PSS/PEI/P3HT:ICBA/EG-PEDOT:PSS) with a PCE of 3.3% under 100 mW cm^−2^ white light illumination. Koppitz et al. reported all-plastic organic solar cells comprising top and bottom PEDOT:PSS electrodes embedded with AgNWs [62]. The devices fabricated in air by doctor-blading exhibit excellent robustness in bending experiments with a PCE of 3.8%. The first all-plastic and all-solution-processed tandem solar cells (up to seven junctions) have been reported by Tong et al. [63] They optimized the conductivity of the charge recombination layers to fabricate multi-junction (up to seven junctions and 22 layers, as shown in Figure 4e,f) solar cells achieving a PCE of 6.1% ± 0.4% and a high *V*_OC_ of 5.37 V. These all-plastic multijunction solar cells are successfully used to drive LEDs and LCDs as well as split water.

### 3.4. Perovskite Solar Cells Based on PEDOT:PSS Electrode

Perovskite solar cells (PeSCs) are the most promising candidates amongst the next-generation photovoltaic technologies and have been attracting considerable attention because of their low fabrication cost and impressive PCE. Undoubtedly, the highly conductive PEDOT:PSS can also be used as a transparent cathode and/or anode in the PeSCs to replace transparent conducting oxides (TCOs) and vacuum-deposited metals. Commonly, the PEDOT:PSS electrode was mainly used as the bottom electrode of TCO-free PeSCs and the top electrode of metal-free PeSCs.

The flexible PeSCs without inorganic TCO layers were successfully fabricated for the first time in 2015. Kelly et al. substituted the metal oxide electrode with a layer of HC-PEDOT:PSS to improve device flexibility of PeSCs. The resulting devices with an architecture of PET/HC-PEDOT/SC-PEDOT/CH_3_NH_3_PbI_3_/PC_61_BM/Al displayed PCEs as high as 7.6% [64]. Ouyang et al. investigated PEDOT:PSS films as the transparent bottom electrode in both rigid and flexible PeSCs. The conductivity of PEDOT:PSS films was significantly enhanced through treating the film with methanesulfonic acid (MSA). The optimized PCEs were 11.0% and 8.6% for the rigid PeSCs-based glass substrate and flexible PeSCs-based PET substrate, respectively [65]. Meanwhile, PEDOT:PSS films with the addition of high boiling polar solvent as an additive were reported as a semitransparent anode for TCO-free flexible PeSCs. The best case in terms of spray deposition (sheet resistance ~28 Ω/sq, transmittance ~65%) was achieved by modifying PH1000 with addition of 6% (v/v) ethylene glycol and a spray deposition time of 90 s [66]. Other environmentally friendly acid-free approaches through solvent additives, post treatments, and a mild oxygen plasma treatment were also presented to enhance the conductivity of PEDOT:PSS for TCO-free PeSCs. The achieved 10.5% was one of the highest reported efficiencies for TCO-free PeSCs with a PEDOT:PSS electrode that excludes the use of acid treatments [67]. Considering the high enough work function of PEDOT:PSS, it can serve as hole transport layer at the same time. Several electrode formulas (PH1000-5%, PH1000-10%, and PH1000-H) were optimized and selected for TCO-free PeSCs without hole transport layer for the purpose of simplifying the process of PeSCs. The electrodes PH1000-x% were obtained from spin-coating the PH1000 solutions containing x vol% DMSO on glass substrates. PH1000-H was obtained from spin-coating DMSO solution of p-toluenesulfonic acid on PH1000. The PeSCs based on PH1000-10% achieved the highest PCE up to 9.65% [68]. In addition to the anode, TCO-free planar PeSCs basing a high conductive PEDOT:PSS as cathode were also successfully fabricated. The polyetherimide (PEI)-modified PEDOT:PSS significantly changed its work function from −5.06 to −4.08 eV, yielding a better electron collection. The PeSCs based on glass/PEDOT:PSS/PEI cathode exhibited an average PCE of 12.42%, comparable to that of regular devices with metal oxide cathodes [69].

**Figure 5:**
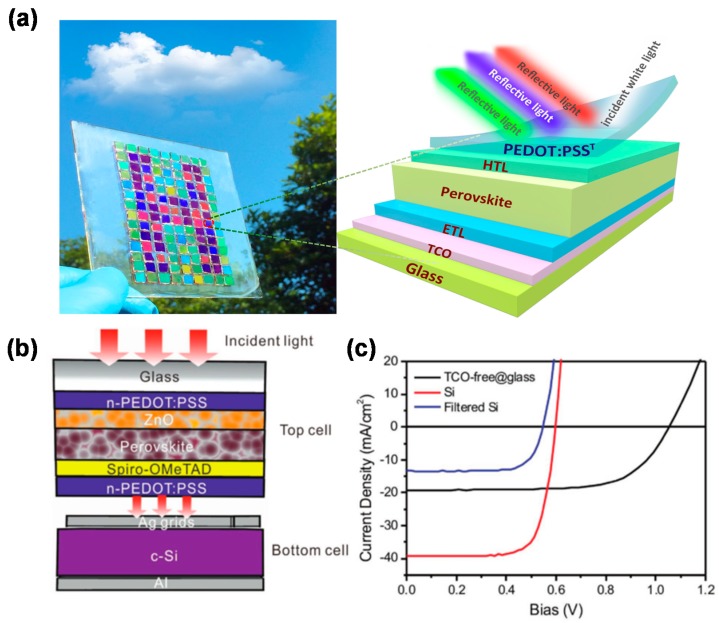
(**a**) Device architecture of the cells with conducting polymer PEDOT:PSS^T^ as the top electrode (left) and photographic image of a colored schematic “H” assembled by colorful PeSCs [75]. Each pixel substrate is about 5 × 5 mm^2^. The PEDOT:PSS electrode was prepared by film transfer lamination. (**b**) Illustration of the structure of the four-terminal TCO-free/c-Si tandem cell and (**c**) the corresponding *J*-*V* curves [77].

Since perovskite is sensitive to humidity, the key problem is that the aqueous solution of PEDOT:PSS destroys the perovskite film when PEDOT:PSS is used as a top electrode for metal-free PeSCs. To solve this incompatibility, film-transfer lamination technique is an appropriate method to prepare PEDOT:PSS film. Jiang et al. employed HC-PEDOT:PSS as the top electrode of PeSCs for the first time through a transfer lamination technique in 2015 [70]. Furthermore, they presented semitransparent PeSCs via the film transfer laminated PEDOT:PSS as the top transparent electrode. The plastic wrap was used as the transfer medium. Transmittance of the fabricated solar cells was tuned by changing the thickness of TiO_2_ scaffold layer and the concentration of PbI_2_ solution. The semitransparent PeSCs yielded a PCE of 10.1% at an area of about 0.06 cm^2^ [71]. A micrometer-thick PEDOT:PSS-Ag nanowire composite with desirable optical, electrical, and adhesive characteristics [72] and mesh-like silver network on PET substrates coated with a PEDOT:PSS/D-sorbitol film were also shown to be a highly transparent top electrode for PeSCs via a simple lamination process [73]. Considering the typically used high-temperature sintered TiO_2_, the mechanically brittle TCO is not suitable for the flexible devices. Zhou et al. demonstrated efficient flexible ITO-free PeSCs. The low-temperature-processed stearyldimethylbenzylammonium chloride (SDBAC)-doped fullerene was used as the electron-transporting layer and silver as the bottom electrode. With the new device structure and the new electron transfer layer, the devices on PES substrates delivered a PCE up to 11.8%, which is comparable to the reference devices on glass substrate [74]. Intriguingly, the top electrode of transparent conducting PEDOT:PSS can be simultaneously used as an spectrally selective antireflection coating. Jiang firstly reported a simple and efficient strategy to achieve colorful PeSCs with wide color gamut by changing the thickness of PEDOT:PSS film and selecting appropriate hole transport layer (Figure 5a). They found the vivid colors arise from the coherent superposition of the reflected and transmitted electromagnetic fields among the transparent PEDOT:PSS electrode, the hole transport layer, and the perovskite layer [75]. It was observed that HC-PEDOT:PSS was usually employed as top anode for regular PeSCs. The PEI-modified PEDOT:PSS with reduced work function can also be employed as top cathode for vacuum-free inverted PeSCs. A polymer stamping with hydrophobic polyurethane acrylate (PUA) succeeded in transferring the both layers of PEDOT:PSS/PEI on top of the ITO/PEDOT:PSS/CH_3_NH_3_PbI_3_/PCBM, improving the device performance from 0.07% to 4.02% [76]. Zheng et al. reported the first low temperature and fully solution-processed TCO-free semitransparent PSCs, in which nitric acid annealed conducting polymer PEDOT:PSS was used as both transparent cathode and anode of the device to replace traditional TCO-based bottom and metal based top electrodes, respectively. The fully solution-processed PeSCs fabricated on glass showed a PCE of 13.9%. In addition, it could readily stack c-Si bottom cell to build a four-terminal PeSCs/c-Si tandem cell (Figure 5a).As shown in Figure 5b, the tandem device showed a high PCE of 19.2% [77].

## 4. Summary and Outlook

Over the past decades, organic polymer thin-film solar cells have made tremendous progress. The device performance increased from roughly 1% to over 17% in less than 30 years. The commercialized conducting polymer PEDOT:PSS endows the PSCs with characteristics of light weight, flexibility, and cost-effectiveness for large-area processing more prominence. It has been reported that the solution-processable PEDOT:PSS can be utilized as bottom, top, and both electrodes to replace traditional non-solution-processed metal oxide and metal electrodes. Despite the great progress of PEDOT-based PSCs, much more work lies ahead to give a comprehensive understanding of its multiple functionalities, resolve outstanding problems, and speed up the research of PEDOT as printing inks for fully printable PSCs. Firstly, the conductivity of solution-processed PEDOT is not enough. How to increase the conductivity of solution-processable PEDOT up to 10^4^ S/cm or even higher is still challenging. Although many strategies have been reported to improve the conductivity of the commercialized PEDOT:PSS, the inner mechanisms are ambiguous. It is necessary to make it clear so that we can solve the problem fundamentally in the initial synthesis step. In addition, the components of the commercialized PEDOT:PSS aqueous dispersion are also unclear. Then, with reference to the device processing, a balance between the transmittance and conductivity also needs to be considered. Especially, the recently emerging high-efficiency non-fullerene active layers possess a wide absorption spectrum extending to near-infrared region. The requirement for the transmittance of PEDOT:PSS electrode needs to be redesigned. Finally, for different processing techniques, new approaches such as surface chemical engineering should also be developed to improve the surface wetting or substrate adhesion.

## Figures and Tables

**Table 1:** Commercial PEDOT:PSS dispersions in water and their properties. (Data are from the previous summarization [8,9]).

Trade Name	Solids Content in Water (wt.%)	PEDOT:PSS Ratio (w/w)	Viscosity at 20 °C (mPa)	Particle Size d_50_ (nm)	Conductivity (S/cm)
Clevios P	1.3	1:2.5	80	80	<10
Clevios PH	1.3	1:2.5	20	30	<10
Clevios PVP AI 4083	1.5	1:6	10	40	10^−3^
Clevios PVP CH800	2.8	1:20	15	25	10^−5^
Clevios PH500	1.1	1:2.5	25	30	500 ^a^
Clevios PH750	1.1	1:2.5	25	30	750 ^a^
Clevios PH1000	1.1	1:2.5	30	30	1000 ^a^

^a^ Conductivities of Clevios PH500, PH750, and PH1000 are measured for dispersions containing 5% dimethyl sulfoxide.

**Table 2:** Summary of the device performance of polymer solar cells (PSCs) based on PEDOT:PSS electrodes under AM1.5 illumination.

Device	Thickness (nm)	R (Ω/sq)	T (%)	*J*_SC_ (mA/cm^2^)	*V*_OC_ (V)	FF	PCE (%)	Ref.
Glass/PEDOT:PSS:S/MEH-PPV/PCBM/Al	150	~10^3^	80	1.6	0.75	0.30	0.36	[3]
Glass/EG-PEDOT:PSS/MEH-PPV:PCBM/Ca/Al	250	250	-	5.1	0.74	0.39	1.5	[28]
Glass/PH500:5%DMSO/P3HT:PC_61_BM/Ca/Al	100	213	90	9.73	0.63	0.54	3.27	[29]
PET/PH500:5%DMSO/P3HT:PC_61_BM/Ca/Al	100	213	90	9.16	0.61	0.50	2.8	[29]
Glass/Methanol treated PH1000/P3HT:PC_61_BM/Ca/Al	~50	25	85	9.51	0.58	0.67	3.71	[30]
Glass/H_2_SO_4_ treated PH1000/PEDOT:PSS(4083)/P3HT:PC_61_BM/Ca/Al	70	67	87	9.29	0.59	0.65	3.56	[20]
Glass/PEDOT:PSS:CNTs/PEIE/ZnO/PBDBTTT-C-T:PC_71_BM/V_2_O_5_-RGO/Ag	-	40.51	80	15.76	0.77	0.62	7.47	[31]
Glass/Ag grid/PH500 /ZnO/C_60_SAM/P3HT:PC_61_BM/PEDOT:PSS(4083)/Ag	-	9.1	79	9.39	0.60	0.57	3.21	[32]
Glass/CH_4_SO_3_ treated PH1000/PEDOT:PSS(4083)/PBDB-T:IT-M/PDINO/Al	80	40	-	16.01	0.925	0.72	10.60	[33]
PET/ CH_4_SO_3_ treated PH1000/PEDOT:PSS(4083)/PBDB-T:IT-M/PDINO/Al	80	40	90	15.49	0.93	0.70	10.12	[34]
Glass/ITO/ZnO/P3HT:PC_61_BM/CPP:PEDOT:PH1000	-	420	-	7.2	0.55	0.58	2.4	[35]
Glass/ITO/PEI/P3HT:ICBA/PH1000:PEG-TmDD	-	526	-	8.70	0.78	0.60	4.1	[14]
Glass/ITO/ZnO/PBDB-T:ITIC/MC-PH1000:EG:PEG-TmDD	-	-	-	13.0	0.86	0.66	7.38	[36]
Glass/metal/ZnO/P3HT:PCBM/PH1000^T^/Ag-busbar	190	-	-	6.96	0.58	0.65	3.08	[37]
Glass/ITO/PEI/P3HT:ICBA/PH1000/PEI/P3HT:ICBA/PH1000:EG:PEG-TmDD ^T^	-	-	-	3.10	1.62	0.68	3.60	[38]
Glass/ITO/PEI/P3HT:ICBA/PEDOT:PSS(4083)/HCT-PEDOT:PSS ^T^	2780	2.60	-	8.65	0.81	0.66	4.6	[39]
Glass/PH500:5%DMSO/ZnO-NPs/C_60_-SAM/P3HT:PCBM/PEDOT:PSS(4083)/PH500:5%DMSO ^T^	130	370	-	5.49	0.31	0.28	0.47	[40]
PES/PH1000 5% DMSO/PEI/P3HT:ICBA/PH1000:CPP-PEDOT ^T^	130 (bottom) 160 (top)	-	-	7.1	0.80	0.52	3.0	[41]
PES/PH1000:5%DMSO/PEI/P3HT:ICBA^T^/PH1000:5%DMSO ^T^	120 (bottom) 150 (top)	-	-	5.6	0.80	0.55	2.4	[42]
Glass/LWF-PEDOT:PSS/P3HT:ICBA/HWF-PH1000:EG:PEG-TmDD ^T^	124 (bottom) 150 (top)	-	-	8.10	0.81	0.61	4.0	[43]
PES/H_3_PO_4_-PEDOT:PSS/PEI/P3HT:ICBA/EG-PEDOT:PSS	85 (bottom) 150 (top)	120 (bottom)	-	6.6	0.84	0.60	3.3	[44]
PES/hc-PEDOT:PSS/PEI/P3HT:ICBA/PEDOT:PSS/PEI/…P3HT:ICBA/hc-PEDOT:PSS^T^	-	-	-	0.40	5.40	0.40	0.85	[45]

Recent representative research progress on PEDOT:PSS-based solar cells including served as bottom, top and both electrodes are included in this table. The parameters, such as thickness, sheet resistance (R) and transmittance are referring to the PEDOT:PSS-based bottom or top electrodes in the PSCs device. The superscript ^T^ of the electrode in the device structure refers to the PEDOT:PSS layer deposited via film transfer lamination technique. CNTs: Carbon nanotubes. *J*_SC_: Short-circuit current density. ***V*_OC:_** open-circuit voltage. FF: Fill factor. PCE: Power conversion efficiency.
